# Lung cancer referral patterns in the former Yorkshire region of the UK

**DOI:** 10.1038/sj.bjc.6600029

**Published:** 2002-01-07

**Authors:** P P Melling, A C Hatfield, M F Muers, M D Peake, C J Storer, C E Round, R A Haward, S M Crawford

**Affiliations:** Northern and Yorkshire Cancer Registry and Information Service (NYCRIS), Arthington House, Hospital Lane, Leeds LS16 6QB, UK; The General Infirmary, Great George Street, Leeds LS1 3EX, UK; Pontefract General Infirmary, Southgate, Pontefract WF8 1PL, UK; Airedale General Hospital, Skipton Road, Steeton, Keighley BD20 6TD, UK

**Keywords:** lung cancer, referral patterns, treatment by specialist, waiting times

## Abstract

The purpose of this study was to find out what proportion of patients are referred as lung cancer guidelines assume, whether different referral pathways result in different management and what proportion of patients are seen within recommended time intervals between referral and treatment. A randomly selected sample of 400 lung cancer cases registered with the former Yorkshire Cancer Registry database in 1993 was selected for casenote analysis. Mode of presentation, speciality of initial referral, treatment by specialist, time intervals for key points in the referral pathways were analyzed. A total of 362 (90.5%) of case-notes were available. Less than half of lung cancer patients (173, 47.8%) presented to hospital with a chest X-ray diagnosis of lung cancer. Forty-one (11.3%) presented as self-referrals to Accident and Emergency and the remainder were referred without a diagnosis of lung cancer by other routes, mainly via GPs. Patients who did not present initially with a lung cancer diagnosis were less likely to receive specialist care (62% : 96%), or have their diagnosis histologically confirmed (57.1% : 80.3%) or receive surgery or radical radiotherapy (6.9% : 13.9%). Nine per cent of all 362 patients did not receive a specialist opinion. Eighty per cent of patients referred by a GP with CXR suspected lung cancer were seen at hospital within 2 weeks. Only 32.4% of those receiving active treatment were treated within 8 weeks of clinical diagnosis or first hospital visit. Lung cancer patients presenting to hospital without a suspicious CXR are less likely to have specialist care, histological confirmation of their cancer and have lower rates of active treatment (surgery, any radiotherapy or chemotherapy).

*British Journal of Cancer* (2002) **86**, 36–42. DOI: 10.1038/sj/bjc/6600029 www.bjcancer.com

© 2002 The Cancer Research Campaign

## 

Lung cancer is the most common cause of cancer death, and over 37 000 cases are diagnosed in the UK every year (Office for National Statistics, ONS, 1998). The median survival in the UK is about 5 months with overall 5-year survival in Yorkshire being less than 6%, rising to 35% in those receiving surgical treatment (Cancer Outcomes Monitoring, 1999) (available at www.nycris. org.uk). Most European countries have higher survival rates (Janssen-Heijnen *et al*, 1998). Prompt referral and good teamwork are essential at every stage of its management. Better organized service delivery coupled with advances in treatment have the potential to significantly improve standards of care.

Lung cancer treatment and referral guidelines (BTS, 1998; NHSE, 1998; Scottish Intercollegiate Guidelines Network, SIGN, 1998; SMAC, 1994) assume that most patients will present to their primary care physician, general practitioner (GP), with chest or respiratory symptoms. The GP will then organize a CXR, and if this suggests lung cancer, patients are then referred to a chest physician with a working diagnosis of lung cancer. Clinical experience suggests that this is not always the case. Using a population based sample of lung cancer patients we wished to identify what proportion of patients were referred by their GP with or without a diagnosis of lung cancer suspected and what proportion were admitted acutely. We examined to whom patients were referred initially and whether they received any specialist management. We examined the time intervals between symptoms, presentation, diagnosis, referral and treatment. Some of these issues have been explored in Scottish patients (Kesson *et al*, 1998; Fergusson *et al*, 1999; Gregor *et al*, 1999, 2001). Kesson *et al* (1998) considered referral times and treatment. Fergusson *et al* (1999) linked treatment rates with seeing a chest physician. Gregor *et al* (1999, 2001) noted variations in management and treatment rates linked to survival in different health board areas. Data from West London showed a low level of specialist management of their lung cancer patients, (Pickles and Rudolf, 1998) but little detail on referral patterns was reported in any of these studies. The purpose of this study was to find out what proportion of patients are referred as lung cancer guidelines assume, whether different referral pathways result in different management and what proportion of patients are seen within recommended time intervals between referral and treatment.

## MATERIALS AND METHODS

The study was designed to provide descriptive information about the referral pathways for lung cancer and, as such, no formal sample size calculations were made. A pilot study (based on 50 patients), undertaken in advance of this study, showed that we could obtain information on investigation, treatment and management from GP and hospital records. It also showed that there were two main presentations of lung cancer (with and without a chest X-ray diagnosis) of roughly equal proportions. An overall sample size of 400 was therefore decided upon, with the aim of obtaining a reasonable number of patients in each presentation group, but also reflecting practical considerations. We took a 400-patient random sample from the former Yorkshire Cancer Registry (population 3.7 million) population based database of 2456 cases of lung cancer registered as being incident in 1993. Exclusion criteria were private treatment or cases whose treatment was organized entirely extra-regionally. Those with missing casenotes were excluded from the analysis.

The sample was stratified by three age groups (<65, 65–75, >75) as referral and treatment patterns were likely to vary with age (Brown *et al*, 1996; Nugent *et al*, 1997; Turner *et al*, 1999) The sample was also stratified by health authority as area of residence might impact on the study (Cancer Outcomes Monitoring, 1999). After stratification cases were randomly selected using an Excel function. The sample sizes (number per health authority in each age band) were chosen to be proportionate to the number of cases presenting in each health authority during the previous 5 years. This ensured, as far as is possible that the population, randomly chosen, was representative of the patients seen in the whole region.

From the sample selected, introductory relevant baseline information was extracted on registration number, name, sex, age, date of birth, GP, main hospital and area of residence from the Cancer Registry database. The Family Health Services Authority and primary managing hospital clinician of each of these patients were contacted and their permission to view the casenotes obtained. GPs were contacted in the case of living patients. Local Research Ethics Committee approval was also sought and obtained for each district.

The following data items were extracted from the case notes: patient details, symptoms, management by GP, management by all consultants at all hospitals involved, management dates, death details and last appointment date. Tumour type was classified as clinical where no histological confirmation of lung cancer was available. Small cell cancer included cases which had either small cell or oat cell histology. All other histological diagnosis were classified as non small cell cancer. Where an important procedure such as bronchoscopy was not recorded, we presumed it was not performed. Management was considered to be specialist if given by a chest physician, thoracic surgeon or oncologist. Chest physicians included those general physicians with a special interest in chest medicine. The consultant chest physicians in the research group identified these chest physicians, with the aid of membership of the regional Thoracic Society.

Definitive treatment was defined as surgery (pneumonectomy or lobectomy), radical radiotherapy (radiotherapy directed at treating lung cancer itself) and chemotherapy. Palliative treatment recorded was palliative radiotherapy (for symptom control only), palliative surgery or best supportive care. The difference between radical and palliative was discerned from the notes. Only one definitive treatment was recorded. Where more than one modality of definitive treatment was given surgery was recorded as the definitive treatment, and radiotherapy and chemotherapy were adjuvant and not recorded in the study. Radical radiotherapy (but not palliative radiotherapy) was considered to be the definitive treatment where chemotherapy was also given. All definitive treatments up to the date of the study were recorded. Only 14 patients (3.9%) were alive at that point. Active treatment was considered to have occurred if any definitive treatment or palliative radiotherapy was given, but not best supportive care only.

Guidelines gave a variety of standards for referral to first hospital visit, and referral to treatment (SMAC, 1994; Scottish Intercollegiate Guidelines Network, SIGN, 1998; BTS, 1998). From these we chose two specifics to report here from these guidelines. The percentage referred within 2 weeks of GP referral, and treatment within 8 weeks of first hospital visit for the with diagnosis group or 8 weeks of clinical diagnosis for the without or acute diagnosis group. Presenting symptoms were noted from the GP casenotes and the principal symptom or symptoms recorded as such. Symptoms written down by the GP such as chestiness, bronchitis or chest infection were amalgamated into one heading of chest infection. Where cases suffered from longstanding lung disease, dating the presentation of the first cancer symptom to the GP, proved difficult. Data were extracted from both sets of casenotes by one trained individual and recorded on case report forms designed specifically for the study. To ensure consistency of recording, two researchers extracted the data from the first 50 notes and the results were compared and found to have less than a 5% difference in recording rate. One of these researchers was responsible for extracting the remainder of the case note data. Analysis was conducted using Access, Excel and SPSS programs. The analysis was mainly descriptive but some comparisons of data were made using χ^2^ tests to check for significant differences (*P*<0.05) between groups and between datasets.

## RESULTS

GP and hospital casenotes were traced for 362 out of 400 patients (90.5%). Eleven patients had neither set of notes. GP notes alone were traced for a further 16 (4%) patients. Eleven patients (2.8%) were excluded because of other reasons, five for being private patients, three for diagnosis prior to 1992, one for initial diagnosis in a foreign country, one for returning to a foreign country in the year of diagnosis and one because of lack of patient consent. The excluded patients were 29.7% female (out of 38 cases) compared to 37.6% female in the 362 cases, and mean age of the 27 years excluded patients with age data was 70.4 years compared to 69.9 in the 362 cases. Characteristics of the study patients were compared with population data from 1993 in the recent Key Sites study report for lung cancer (Cancer Outcomes Monitoring, 1999) which provides data on all lung cancer registrations in Yorkshire for the time period 1986–1994. This comparison found that there were no statistically significant differences (*P*>0.05) in patient characteristics between the two sets of patients.

### Mode of presentation

The pilot study noted two main modes of presentation for lung cancer patients and one less common presentation. The same categorization was used for the main study. The ‘With CXR Diagnosis’ group consisted of patients who presented to their GP with a respiratory related complaint. A GP requests a CXR and, on the basis of an abnormal result refers the patient to hospital. There were 173 patients (47.8%) in this group. Patients in the ‘Without CXR Diagnosis’ group were referred to hospital because of their symptoms but with no prior CXR. There were 148 patients (40.9%) in this group, 49 being admitted on day of referral. The ‘Acute’ group presented themselves to Accident and Emergency. There were 41 patients (11.3%) in this group. Patient characteristics are seen in Table 1

**Table 1:**
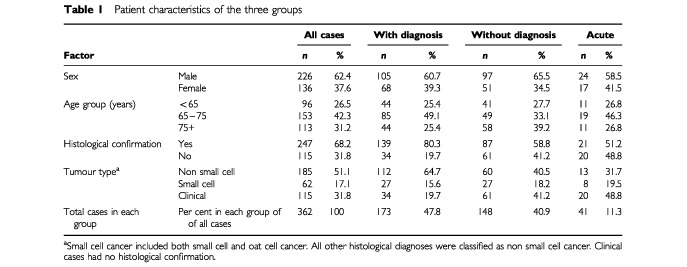
Patient characteristics of the three groups

.

There is some variation between the groups in age, sex and tumour type. Overall there are nearly twice as many men than women. The without diagnosis group has a lower percentage of 65–75-year-olds, and a higher percentage of over 75s, but this difference in age groups is not significant (*P*=0.06). The rates of histological confirmation were far lower in the without diagnosis and acute groups than were the with diagnosis group. There were slightly more small cell cancers in the acute and without diagnosis groups. The proportions in the three groups were significantly different (*P*<0.001) for histological confirmation and tumour type. This difference between the with and without group became of borderline significance when the clinically diagnosed tumour types were removed (*P*=0.052).

### Principal presenting symptoms

One hundred and thirty-nine (80%) of the with diagnosis group presented to their GP with mainly lung related symptoms (cough, chest pain or infection, haemoptysis or dyspnoea) compared to 69 (46.6%, CI: 38.4%, 55.0%) of those without a diagnosis. See Table 2

**Table 2:**
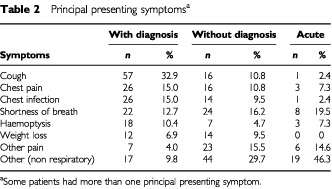
Principal presenting symptoms^a^

for principal presenting symptoms. This lists a patient's main one or two symptoms, but does not exclude them from having other symptoms as well. Only 16 (39%, CI: 24.2%, 55.5%) of the acute group were admitted with a respiratory complaint.

### Investigation and treatment

Variation in both investigations and treatment are shown in Table 3

**Table 3:**
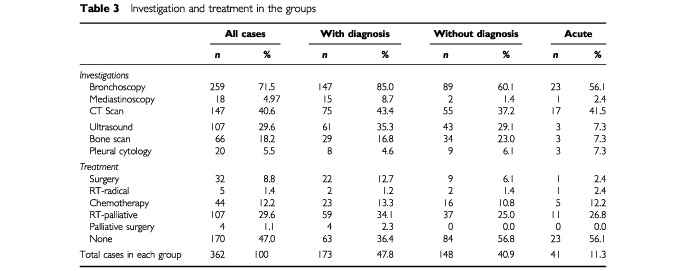
Investigation and treatment in the groups

. Rates of bronchoscopy, mediastinoscopy, surgery and ‘no treatment’ were significantly different (*P*<0.001, *P*=0.008, *P*=0.04, *P*=0.001 respectively) between the three groups. Bronchoscopy and mediastinoscopy were much more common in the with diagnosis group. Other investigations or pleural cytology were more common in different groups. The with CXR diagnosis had more ultrasound, the without diagnosis group had more bone scans and the acute group more pleural cytology. These differences were significant for ultrasound only (*P*=0.002 between all groups, *P*=0.023 when without diagnosis and acute groups are combined)

Surgery, chemotherapy, and palliative radiotherapy were all used most frequently in the with CXR diagnosis group, but the difference was only significant for surgery (*P*=0.035). The number of patients receiving radical radiotherapy was too small to make meaningful comparisons. Treatment was associated with histological confirmation and tumour type. A total of 40 of 62 (64.5%) patients with small cell cancer received chemotherapy, whilst 35 of 185 (18.9%) non small cell cancer patients received surgery or radical radiotherapy. Only two of 115 (1.7%) patients without histological confirmation received chemotherapy or radical radiotherapy, and 29 (25.2%) palliative radiotherapy.

### Specialist management

Table 4

**Table 4:**
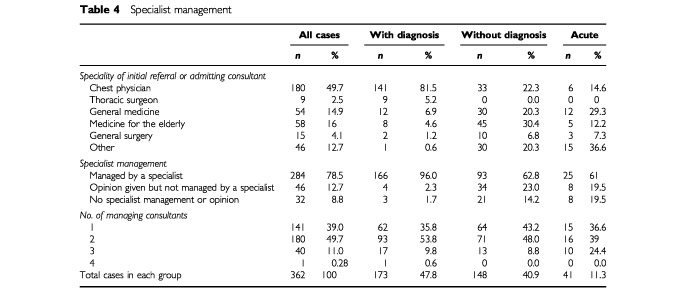
Specialist management

shows specialist management in the three groups. Only half of all the patients were referred initially to a chest physician. Only six (14.6%) of acute patients were admitted to the care of a chest physician (possibly consistent with the chance of an acute medical rota), whereas 141 (81.5%) of those with a diagnosis were referred to a chest physician and a further nine (5.2%) to a thoracic surgeon. Two (1.4%) patients of the without diagnosis patients were initially referred to a medical oncologist.

Overall 284 (78.5%) of patients eventually had specialist care and 78 (21.5%) did not. In the with diagnosis group 166 (96%) had such care whereas only 118 (62%) of the other two groups did. Opinions were sought from specialists in a further 34 (23%) and eight (20%) of without diagnosis and acute cases respectively. Twenty-nine (15%) of the without diagnosis and acute groups had no specialist care.

**Figure 1:**
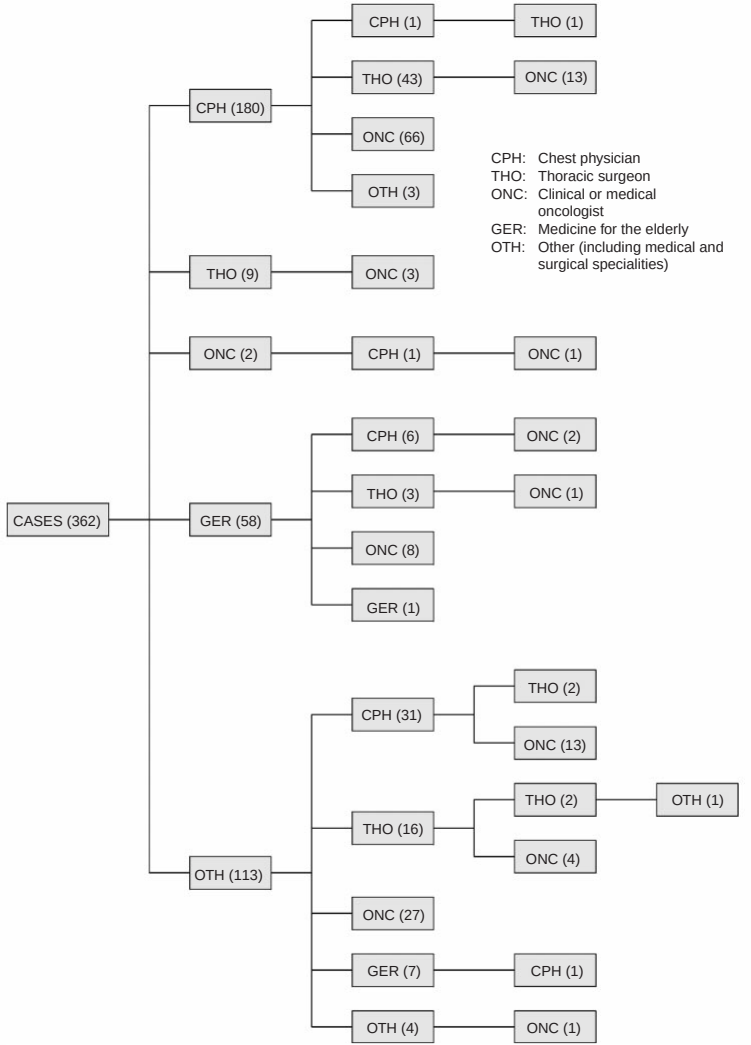
Consultant referral pathways.

A total of 141 (39%) of cases were managed by one consultant. The mode was two consultants and only 40 (11%) of patients were managed by three. There were 70 different pathways of referral to the final consultant. A total of 180 (50%) of cases first saw a chest physician, 63 (35% of them) were then referred to a clinical oncologist, 43 (24%) to a thoracic surgeon, three (2%) to medical oncologists and four (2%) to various other specialties. Most referrals to a third consultant involved clinical oncologists, or thoracic surgeons when the first consultant was not a specialist in lung cancer. Figure 1

and Table 5

**Table 5:**
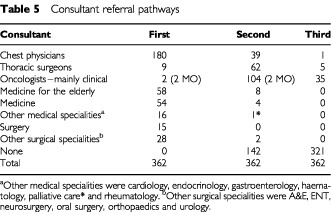
Consultant referral pathways

summarize the pathways.

### Referral and management intervals

Table 6

**Table 6:**
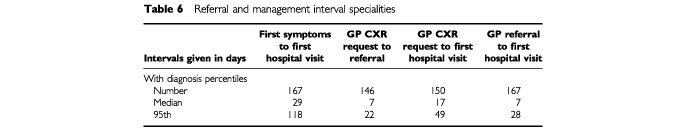
Referral and management interval specialities

shows referral and management interval percentiles in the three groups. Fifty per cent of patients in the with diagnosis group were referred within 1 week of a CXR request and had their first hospital visit within 17 days. Eighty per cent (134) of the with diagnosis patients were seen within 2 weeks of referral by a GP. Seventy-one per cent (55 of 78) of those referred by a GP without diagnosis who were not admitted as an emergency on that day were seen within 2 weeks. Median time from first visit to any treatment was 35 days in the with diagnosis group and median time from clinical diagnosis to treatment was 28 days for the without diagnosis group (see Figure 2

**Figure 2:**
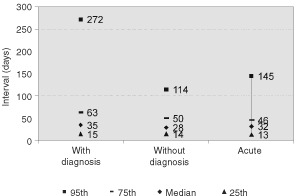
Percentile intervals between first hospital visit (or clinical diagnosis) and treatment.

). Decisions not to treat were made more quickly in all groups, with a median of 17 days when recorded in the notes. Thirty-seven patients (10.2%) received definitive treatment. Only 37.5% (9 of 24) of the with diagnosis group received definitive treatment within 8 weeks of first hospital visit and only 23.1% (3 of 13) of the acute and without diagnosis group were treated within 8 weeks of clinical diagnosis. The difference between the groups is not significant (*P*=0.515).

## DISCUSSION

We have found no previous population-based studies looking directly at referral patterns for lung cancer despite most guidelines assuming a referral pathway to hospital that includes a CXR ordered by a general practitioner. We found that less than 50% of patients present in this fashion. A similar sample of patients in Glasgow found 57% were initially referred to chest physicians including some in whom the diagnosis was incidentally found on chest X-ray (Kesson *et al*, 1998). An all Scotland study found 58% referred initially to chest physicians which is higher than in our data (Gregor *et al*, 1999). Neither of these studies considered referral pathways and CXR use in detail. One local UK audit noted the dispersal of patients to many disciplines (Pickles and Rudolf, 1998), and a Royal College of Physicians audit noted that 62% of patients were referred by GPs to chest physicians, and that these patients had better survival at 6 months (Thompson *et al*, 1999). However the latter study was confined to patients who underwent bronchoscopy. Surgical treatment was only 5% in the Kesson study (Kesson *et al*, 1998), but 11.6% in the later Gregor study (Gregor *et al*, 1999, 2001), compared to 8.8% in our study and 10.6% in the wider Yorkshire study (Cancer Outcomes Monitoring, 1999). In comparison to our 47% with no active treatment, and 48.1% in the all Yorkshire study, 38.4% of the Gregor study had supportive care only (with a further 20% with unknown management), and 42.2% received no active treatment in the Gregor study. It is likely that our results are generalizable across the UK but not necessarily to other countries. Our stratified sample of 400 (362 cases with both casenotes) comes from a population based cancer registry. The diversity of the Yorkshire population helps to ensure generalizability. Our sample is comparable in age, sex, district of residence, histology and treatment to that involving an 8-year retrospective study of lung cancer from the same database using a total of 22 000 cases, 2456 of which were registered in 1993 (Cancer Outcomes Monitoring, 1999).

Different ways of presentation to hospital are associated with different rates of investigations, treatment and management of patients. Variation in investigation and treatment rates have been noted previously but not compared with routes of referral (Fergusson *et al*, 1996; Gregor *et al*, 1999; Richardson *et al*, 2000) The proportion of patients without a histological diagnosis has been proposed as an indicator of the proportion of patients not amenable to optimal treatment, which can be used to compare different registry populations (Crawford and Atherton, 1994). It is not certain to what extent these different types of management are due to variation in casemix. The three groups had no significant differences in age and sex but tumour types were significantly different. This difference in tumour type could be due to casemix with some tumours such as small cell presenting more acutely and less in the classical manner. It may be due to the significantly different and lower histological confirmation rates in the acute and without groups, but histological confirmation is likely to be lower in cases with poor functional status. Fergusson *et al* (1999) found patients referred to a chest physician were younger and at an earlier stage. We have little information on stage of disease or co-morbidity at presentation. The numbers of patients recorded to have distant metastases in each group are not significantly different but recording of metastases in casenotes is poor, particularly those metastases occurring in lymph nodes, and many patients are in the unknown category. Lower surgery rates might suggest that the without diagnosis and acute group presented later, but the with diagnosis group also has higher rates of palliative radiotherapy. An alternative possibility is that investigation and treatment differed because these patients had less specialist care than the with diagnosis group (62% compared to 96%). In three previous studies radical treatments were more likely in those reviewed by a chest physician (Brown *et al*, 1996; Muers and Haward, 1996; Fergusson *et al*, 1999).

The referral times varied widely between cases and groups. Reassuringly 80% of GP referred patients with a CXR diagnosis were seen within 2 weeks, long before the current 2 week initiative (DoH, 1997, 1999). This is similar to the findings of a retrospective survey of English Trusts using 1997 data, (Spurgeon *et al*, 2000), and with the Glasgow study (Kesson *et al*, 1998). Of concern is that 67.6% of patients given radical treatment (surgery and radical radiotherapy) did not receive this within the suggested guideline (Scottish Intercollegiate Guidelines Network, SIGN, 1998; SMAC, 1994) time of within 8 weeks of their first hospital visit (with diagnosis group) or within 8 weeks of clinical diagnosis (other groups); but this data does predate these guidelines. This interval is longer than in the retrospective survey (Spurgeon *et al*, 2000) which has a median time of 39 days, but the numbers of lung cancer cases for 1 month in England are less than expected in that study. Decisions not to treat were taken quickly. It is not certain what caused these delays to treatment. It may be that investigations were slow in arrangement or reporting or that there were long waiting lists for suitable treatment such as radical radiotherapy or surgery. Long times to treatment have been noted by others (Billing and Wells, 1996; Dische *et al*, 1996; Kesson *et al*, 1998). The delays experienced by lung cancer patients are long in relation to the short median survival of 5 months of these patients (Cancer Outcomes Monitoring, 1999), and longer than those for most other common cancers. Further detail on the study may be found in the full NYCRIS report (Cancer Outcomes Monitoring, 2000).

There is a common assumption that lung cancer patients present to hospital with a CXR suspicious of lung cancer. We found that over 50% of cases did not present this way. Clinicians in hospital and general practice should be more aware of the diversity of presentations of lung cancer. All patients with suspected lung cancer should be referred to a member of the lung cancer team, usually a chest physician. The intervals between management events such as time to referral or treatment should be audited and avoidable delays reduced to a minimum. It is of concern that lung cancer patients presenting to hospital without a suspicious CXR are less likely to have specialist care, histological confirmation of their cancer and that they have lower rates of active treatment (surgery). Whether this is because of more advanced disease or their different patterns of care urgently needs to be determined.
